# Emergent Synchronous Bursting of Oxytocin Neuronal Network

**DOI:** 10.1371/journal.pcbi.1000123

**Published:** 2008-07-18

**Authors:** Enrico Rossoni, Jianfeng Feng, Brunello Tirozzi, David Brown, Gareth Leng, Françoise Moos

**Affiliations:** 1 Department of Computer Science, University of Warwick, Coventry, United Kingdom; 2 Centre for Computational System Biology, Fudan University, China; 3 Department of Physics, University of Rome ‘La Sapienza’, Rome, Italy; 4 The Babraham Institute, Cambridge, United Kingdom; 5 Centre for Integrative Physiology, University of Edinburgh, Edinburgh, United Kingdom; 6 Biologie des Neurones Endocrines, Montpellier, France; University College London, United Kingdom

## Abstract

When young suckle, they are rewarded intermittently with a let-down of milk that results from reflex secretion of the hormone oxytocin; without oxytocin, newly born young will die unless they are fostered. Oxytocin is made by magnocellular hypothalamic neurons, and is secreted from their nerve endings in the pituitary in response to action potentials (spikes) that are generated in the cell bodies and which are propagated down their axons to the nerve endings. Normally, oxytocin cells discharge asynchronously at 1–3 spikes/s, but during suckling, every 5 min or so, each discharges a brief, intense burst of spikes that release a pulse of oxytocin into the circulation. This reflex was the first, and is perhaps the best, example of a physiological role for peptide-mediated communication within the brain: it is coordinated by the release of oxytocin from the dendrites of oxytocin cells; it can be facilitated by injection of tiny amounts of oxytocin into the hypothalamus, and it can be blocked by injection of tiny amounts of oxytocin antagonist. Here we show how synchronized bursting can arise in a neuronal network model that incorporates basic observations of the physiology of oxytocin cells. In our model, bursting is an emergent behaviour of a complex system, involving both positive and negative feedbacks, between many sparsely connected cells. The oxytocin cells are regulated by independent afferent inputs, but they interact by local release of oxytocin and endocannabinoids. Oxytocin released from the dendrites of these cells has a positive-feedback effect, while endocannabinoids have an inhibitory effect by suppressing the afferent input to the cells.

## Author Summary

When young suckle, they are rewarded intermittently with a let-down of milk that results from reflex secretion of the hormone oxytocin. Oxytocin is a neuropeptide made by specialised neurons in the hypothalamus, and is secreted from nerve endings in the pituitary gland. During suckling, every 5 min or so, each of these neurons discharges a brief, intense burst of action potentials; these are propagated down the axons, and release a pulse of oxytocin into the circulation. Here, we have built a computational model to understand how these bursts arise and how they are synchronized. In our model, bursting is an emergent behaviour of a complex system, involving both positive and negative feedbacks, between many, sparsely connected cells. The oxytocin cells are regulated by independent afferent inputs, but they interact by local release of oxytocin and endocannabinoids. Oxytocin released from the dendrites of these cells has a positive-feedback effect, while endocannabinoids have an inhibitory effect by suppressing the afferent input to the cells. Many neurons make peptides that act as messengers within the brain, and many of these are also released from dendrites, so this model may reflect a common pattern-generating mechanism in the brain.

## Introduction

The milk-ejection reflex is perhaps the best example of a physiological role for peptide-mediated communication within the brain. Here we use a large body of data, accumulated over the last 30 years, to develop a model of this reflex. In the model, synchronized bursting is an emergent property of the network; we use the model to explain diverse experimentally observed phenomena, many of which seem paradoxical.

When young suckle, they are rewarded intermittently with a let-down of milk that results from the reflex secretion of oxytocin [1]. Oxytocin is made in about 9,000 magnocellular neurons, each of which sends a single axon to the posterior pituitary, where it gives rise to about 2000 neurosecretory varicosities. From these varicosities, large vesicles that contain oxytocin are secreted by exocytosis [2] in response to action potentials (spikes), propagated down the axons [3]. Normally, oxytocin cells discharge asynchronously at 1–3 spikes/s, but during suckling, every 5 min or so, they all discharge a brief burst of spikes (50–150 spikes in 1–3 s) that releases a pulse of oxytocin [4]; this pulse, travelling in the systemic circulation, causes cells of the mammary gland to release milk into a collecting duct from which it is extracted by suckling.

In lactating rats, the background activity of oxytocin cells is like that in non-lactating rats; the cells fire slowly, asynchronously and nearly randomly. Suckling produces little change in this except that slow firing cells tend to speed up slightly, while faster firing neurons slow down. After a few minutes, the first bursts occur; these are small and involve only some cells, but progressively more cells are recruited until all show intense bursts [5]. Bursts are elicited by suckling, but not by most other stimuli; for example, systemic injections of cholecystokinin produce an increase in electrical activity that is identical in lactating and non-lactating rats, and which consists of a steady increase in firing rate that persists for 10–15 min [6].

Milk-ejection bursts vary in size from cell to cell and according to the strength of the suckling, but are consistent in their overall shape, especially from one burst to the next in any given cell. These features [7],[8] led to the belief that bursting reflects mechanisms intrinsic to oxytocin cells, but these mechanisms have proved elusive. Whole-organ cultures of neonatal rat hypothalamus display networks of oxytocin cells that burst periodically [9]; these bursts are synchronized, but inter-burst activity also shows high levels of synchrony, unlike *in vivo* observations, and the bursts are generally longer and less intense than milk-ejection bursts. Oxytocin cells in slice preparations also display bursts when maintained in low extracellular [Ca^2+^] and exposed to phenylephrine, but these are not synchronized, and are less intense than milk-ejection bursts *in vivo* [10]. With these partial exceptions, *in vitro* preparations have not reproduced the bursting seen *in vivo*, indicating that it depends on unknown features of the suckling input.

The two supraoptic nuclei contain about 2000 of these oxytocin cells and (in virgin rats) about 3.2 ng of oxytocin, about 95% of which is in the dendrites [11]. Oxytocin cells have 2–5 dendrites, several hundred micrometres long, which are filled with neurosecretory vesicles that can also be released by exocytosis [12]. In a virgin rat, each cell has >10,000 vesicles in its dendrites [12], each vesicle containing ∼85,000 molecules of oxytocin [11], and in lactating rats, oxytocin synthesis is further elevated [13]. The cells intercommunicate within “bundles” of 3–8 dendrites; in lactating rats, these bundles are encapsulated by glial processes, but within a bundle the dendrites are directly apposed to each other [4],[14],[15].

Dendritic oxytocin release in basal conditions *in vivo* is not much influenced by spike activity, but can be evoked by stimuli that mobilize intracellular Ca^2+^ [16]. When oxytocin is released, it acts at high-affinity receptors on the dendrites [17] to depolarize oxytocin cells [18]; it also mobilizes Ca^2+^ from intracellular stores [19], which promotes the further release of oxytocin [20]. The mobilisation of Ca^2+^ has another important consequence: it can “prime” the dendritic stores of oxytocin, making them available for subsequent activity-dependent release [21]. We have suggested that the suckling input might prime the dendritic stores of oxytocin, making them available for activity-dependent release [21], and that this is essential for bursting. During suckling, dendritic oxytocin release is detected *before* any increase in the electrical activity of oxytocin cells, and before any increase in pituitary secretion [22]. Central injections of oxytocin facilitate bursting in the presence of suckling, but are ineffective in its absence; conversely, local injections of oxytocin antagonists block suckling-induced bursting [23]. Oxytocin cells also modulate afferent inputs via the production of endocannabinoids (and other substances), which inhibit excitatory inputs presynaptically [24], and oxytocin suppresses inhibitory inputs by attenuating the effects of GABA [25]. Oxytocin also acts on glial cells to promote morphological reorganization that facilitates dendro-dendritic interactions [14],[15].

Here we show that bursting can arise as an emergent property of a model network constructed to incorporate the observations summarized above.

## Model

Each model neuron is a modified leaky integrate-and-fire model subject to stochastic excitatory and inhibitory postsynaptic potentials. The modifications include a post spike relative refractoriness that mimics the hyperpolarising afterpotential (HAP) that follows single spikes in oxytocin cells [26]. This is modelled as a transient rise in spike threshold, and reproduces the distribution of interspike intervals *in vivo*, which is largely determined by the HAP [27]. Another modification mimics the effect of a slower activity-dependent afterhyperpolarisation (AHP); this sustains a prolonged reduction in excitability after intense activation, and is enhanced in oxytocin cells in lactation [28]. In the model, dendritic release is coupled to spike activity non-linearly; oxytocin secretion from the pituitary is non-linear in that there is a marked facilitation of secretion at high spike frequencies [29], and we assume that dendritic release is similarly facilitated [30]. Dendro-dendritic interactions are modelled by elements that mimic the excitatory actions of oxytocin (implemented as an activity-dependent reduction in spike threshold) and the autocrine effects of endocannabinoids which feed back to modulate synaptic input rates.

### Network Topology

**Figure 1:**
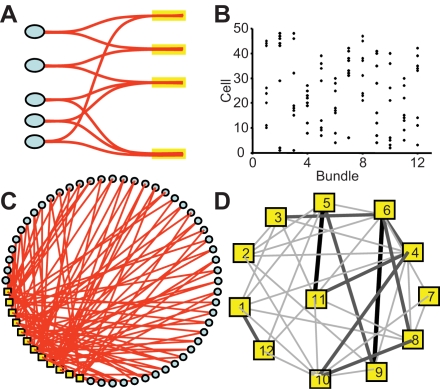
Structure of the Model Network. (A)Schematic diagram of the organization of the oxytocin network; the yellow boxes represent dendritic bundles. (B) The (bipartite) adjacency matrix for a randomly generated network with 48 neurons and 12 bundles; the squares mark non-zero matrix elements. (C) Visualization of the network with blue circles for neurons and yellow squares for bundles. (D) The heterogeneity of connectivity in a randomly wired model of 12 bundles. The width of an edge between any two bundles represents the number of neurons having dendrites in both bundles. In this example, most bundles are ‘bridged’ by at most one neuron; a few others share two or three neurons. By means of such neurons, any increase of the spike activity of the neurons projecting to one bundle can affect the neurons in all the connected bundles, hence rapidly propagates through the network.

A key element of our model is the topology of network connections, which differs from all other topologies of biological networks in the literature. The network has *n* neurons and *n_b_* bundles, and each neuron has two dendrites in different bundles [4],[14],[15]. The network can be described by a bipartite graph *G* = {*N*∪*B*, *E*}, where *N* is the set of neurons, *B* the set of bundles, and *E* the set of connections from neurons to bundles such that, for a neuron *a* ∈ *N* and a bundle *b* ∈ *B*, (*a* ,*b*) ∈ *E* if *a* has a dendrite in *b*. The network topology is thus specified by the adjacency matrix **O** = {*o_ij_*}, *i* = 1,…,*n*, *j* = 1,…,*n_b_*, where *o_ij_* = 1 if neuron *i* has a dendrite in bundle *j*, and *o_ij_* = 0 otherwise. If dendro-dendritic connections are formed at random, then **O** is a random binary matrix whose rows satisfy 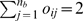. Figure 1 shows such a matrix for a network of 48 neurons and 12 bundles. We considered two procedures in order to assign dendrites to bundles. In both cases, for a network of *n* neurons, and a given integer *d*>0, we start with an empty adjacency matrix of *n* rows and 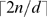 columns. Then, for each neuron we select two bundles as follows. The index of the first bundle *i*_1_ is selected uniformly at random in the set {1,2,…,*n_b_*}, the second index is selected uniformly at random in the set {1,2,…,*n_b_*}/{*i_1_*}, ensuring that no neuron has two dendrites in the same bundle. For the first procedure, this selection is repeated for all neurons, leading to a completely random allocation of dendrites into bundles. There is a finite probability that some bundles are never selected, and these are removed from the network. In the second procedure, we keep track of the number of dendrites in each bundle and, once one bundle contains *d* dendrites, this is excluded from further selection. This we refer to as a “homogeneous arrangement of the connections” as each of the *n_b_* bundles contains the same number of dendrites.

#### Model of single neuron

**Figure 2:**
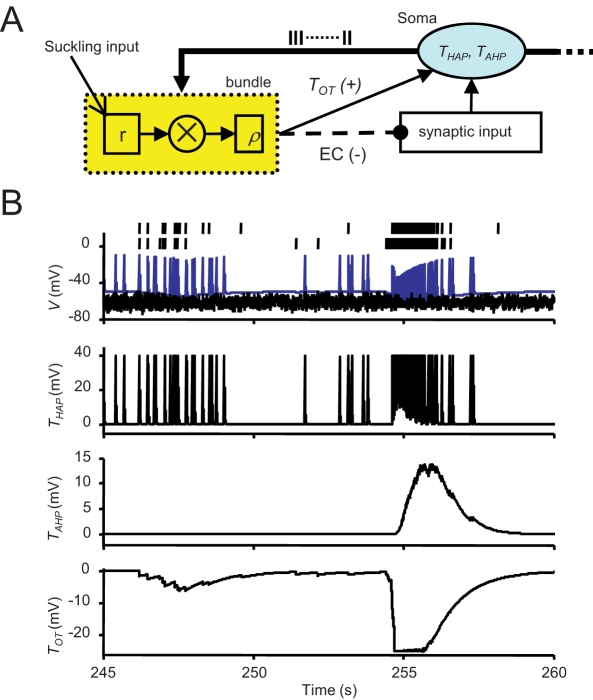
The Structure of a Single Model Neuron. (A) Schematic illustrating the organization of a single model neuron: it receives random excitatory and inhibitory synaptic inputs, and its excitability is modelled as a dynamically changing spike threshold that is influenced by a post-spike HAP (parameter THAP), and a slower AHP (TAHP). Each neuron interacts with neighbouring oxytocin neurons by two dendrites that project to bundles (yellow), and its excitability is increased when oxytocin is released in the vicinity of these dendrites (TOT). Activity-dependent production of endocannabinoids (EC) feeds back to reduce synaptic input rates. (B) This analyses the behavior of one model cell during a burst in detail. The upper two raster traces show the times of occurrence of all oxytocin release events in the two dendritic bundles to which the cell is connected. Below this is the soma activity: the black line (V) shows the impact of excitatory and inhibitory inputs, and the blue line shows the dynamic spike threshold, showing the effects of post-spike activity changes and the effects of oxytocin. The bottom three traces show THAP , TAHP, and TOT.

To model spike generation, we use the leaky integrate-and-fire model, modified to incorporate activity-dependent changes in excitability (Fig. 2). The membrane potential *ν_i_* of cell i obeys
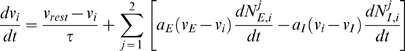
(1)Where τ is the membrane time constant, *ν*_rest_ is the resting potential, 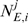, 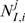 are inhomogeneous Poisson processes of rate 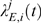, 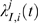, *a_E_*(*ν_E_*−*ν_rest_*), *a_I_*(*ν_rest_*−*ν_I_*), are the magnitude of single EPSPs and IPSPs at *ν_rest_*, and *ν_E_*, *ν_I_* are the excitatory and inhibitory reversal potentials. A spike is produced in cell *i* at time 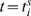, *s* = 1,2,…, , if 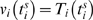, where *T_i_*(*t*) is the spike threshold at time *t*. After a spike, *ν_i_* is reset to *ν_rest_*. Activity-dependent changes in excitability and the effects of oxytocin are modelled by effects on spike threshold:
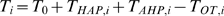
(2)where *T*_0_ is a constant. *T_HAP_* models the effect of a HAP by
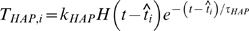
(3)where *k_HAP_*, *τ_HAP_*, are constants, 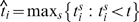, and *H*(*x*) is the Heaviside step function. This gives a transient increase in spike threshold after each spike. *T_AHP_* models the effect of the AHP. The AHP builds up slowly, leading to a significant reduction of excitability only after relatively intense activity. The variables *f_i_*, *i* = 1,…,*n*, represent the recent activity of each neuron, and
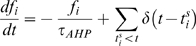
(4)where *τ_AHP_* is the decay constant of the AHP, and δ(*x*) is the Dirac delta function. We set
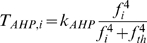
(5)where *k_AHP_*, *f_th_* are constants adjusted to match the known characteristics of spontaneous firing in oxytocin cells. The increase in excitability due to oxytocin is modelled by *T_OT_*,
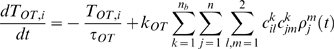
(6)where *τ_OT_*, *k_OT_* are constants, 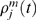 is the instantaneous release rate from dendrite *m* of cell *j*, and the sums pick up all the cells whose dendrites share the same bundle as cell *i*. The network topology is represented by matrices 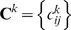, *k* = 1,…,*n_b_*; 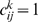 if dendrite *j* of cell *i* is in bundle *k*, and zero otherwise. To model saturation of the oxytocin receptors, the oxytocin-dependent reduction of the spike threshold is limited to a maximum (*T_OT,max_*) of 25 mV.

The readily-releasable store of oxytocin (the store accessible by activity-dependent release) in dendrite *j* of cell *i* is represented by 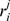, where
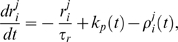
(7)where *τ_r_* is a time constant, *k_p_*(*t*) is the rate of priming due to the suckling input (*k_p_*(*t*) is a positive constant during suckling and zero otherwise), and 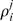 is the instantaneous release rate from dendrite *j*. Release is proportional to the readily-releasable stores, so
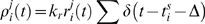
(8)where *k_r_* is the maximum fraction of the stores that can be released by a spike, Δ is a fixed delay before release, and the summation extends over the set 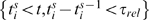, with *τ_rel_* a constant. This ensures that only spikes occurring at intervals of less than *τ_rel_*, (i.e. instantaneous firing rates exceeding 1/*τ_rel_*) induce any release from dendrites. In the model, we set *τ_rel_* = 50 ms, corresponding to an instantaneous firing rate threshold for release of 20 Hz, but the exact value is not critical.

The variables *ε_k_* (*t*), *k* = 1,…,*n_b_* represent the concentration of endocannabinoids in each bundle, and evolve according to
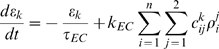
(9)where *τ_EC_* is the decay time constant, and *k_EC_* scales the amount of oxytocin released within the bundles into an increase of endocannabinoid concentration. Implicitly, we assume that endocannabinoids are produced in oxytocin cells as a consequence of the mobilisation of intracellular Ca^2+^ that occurs in response to oxytocin. For simplicity, we assume that the rates of both excitatory and inhibitory synaptic inputs are equally affected by endocannabinoids [24], and neglect the direct effect of oxytocin on the actions of GABA [25] as duplicated by this. Thus
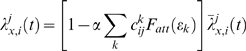
(10)where 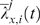, *x* = *E*, *I* are the unmodified synaptic input rates for dendrite *j* of neuron *i*, *α* is the maximal fractional attenuation of the input, and
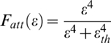
(11)with ε*_th_* constant. The parameter values for simulations are as in Table 1 unless otherwise stated. The equations were integrated numerically with the Euler-Maruyama method using a time step of 0.1 ms. A MATLAB code for simulating the system is available at http://www.informatics.sussex.ac.uk/users/er28/otnet/, see also Video S1).

**Table 1:** The Model Parameters Used for Simulations (a.u., arbitrary units).

Name	Description	Value	Units
*N*	Number of cells	48	
*n_b_*	Number of bundles	12	
*τ*	Membrane time constant	10.8	ms
*ν_rest_*	Resting potential	−62	mV
*a_E_*(*ν_E_*−*ν_rest_*)	EPSP amplitude	4	mV
*a_I_*(*ν_rest_*−*ν_I_*)	IPSP amplitude	4	mV
*ν_E_*	EPSP reversal potential	0	mV
*ν_I_*	IPSP reversal potential	−80	mV
*λ̅* *_E_*	Excitatory input rate	80	Hz
*λ̅* *_I_*	Inhibitory input rate	80	Hz
*k_HAP_*	HAP, maximum amplitude	40	mV
*τ_HAP_*	HAP, decay time constant	12.5	Ms
*k_AHP_*	AHP, maximum amplitude	40	mV
*τ_AHP_*	AHP, time constant	2	s
*f_th_*	AHP, half-activation constant	45	a.u.
*τ_OT_*	Time decay of oxytocin-induced depolarization	1	s
*k_OT_*	Depolarization for unitary oxytocin release	0.5	mV
Δ	Time delay for oxytocin release	5	ms
*T_OT, max_*	Maximum oxytocin-induced depolarization	25	mV
*k_p_*	Priming rate	0.5	s^−1^
*τ_r_*	Time constant for priming	400	s
*k_r_*	Fraction of dendritic stores released per spike (max)	0.045	
*τ_EC_*	Time constant for [EC] decay	6	s
*k_EC_*	Endocannabinoid increase per unit oxytocin release	0.0025	a.u.
*ε_th_*	[EC] threshold for synaptic attenuation	0.03	a.u.
*τ_rel_*	Maximum interspike interval for release	50	ms
*α*	Fractional attenuation of synaptic input rate (max)	0.6	

## Results

**Figure 3:**
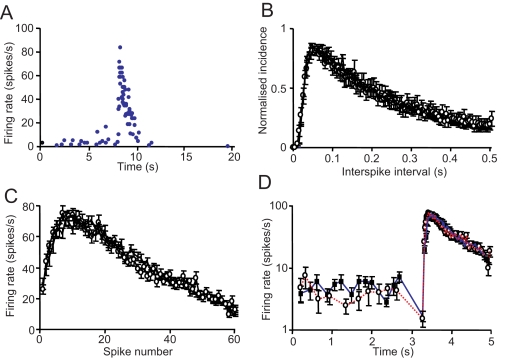
Comparison of Bursting Activity in Real and Modelled Oxytocin Cells. (A) A typical burst in a model cell plotted as instantaneous firing rate (each point is the reciprocal of the interval since the previous spike). This profile is essentially indistinguishable to burst profiles observed in vivo. (B) Consensus interspike interval distribution (see [34]) of 23 oxytocin cells recorded from the supraoptic nucleus in vivo (circles) compared with that generated by the model (squares). In both cases, histograms were constructed from spike activity between the bursts. The individual distributions were normalized to the height of the mode and averaged; bars are S.E.M. (C) Mean profiles of milk-ejection bursts from a real oxytocin cell (circles) and from a model cell (squares). Each profile is constructed from 17 bursts, and shows the mean+S.E. instantaneous firing rate plotted for each interspike interval within the bursts. (D) Mean instantaneous firing rates vs. time of occurrence on a semi-log plot from a real oxytocin cell (circles, red dashed line) and from a model cell (squares, blue line). The semi-log plot displays more clearly the effect on instantaneous frequency just before a burst, and it shows that, in both real cells and model cells, most bursts begin with a slight decrease in instantaneous firing rate. In the model this is because most cells are usually follower cells - a burst has begun elsewhere, and the first indication of this is a decrease in synaptic input as a result of the inhibitory effects of cannabinoids. The bursts begin only when the excitatory effect of oxytocin exceeds this.

We show simulations from a network of 48 neurons and 12 bundles (mean number of dendrites per bundle *d̅* = 2*n*/*n_b_* = 8) with the topology as in Fig. 1B. We have also simulated larger networks (*n* = 3000, *d̅* = 8), and all the results reported below remain qualitatively similar. The network displays synchronized high-frequency bursts (Fig. 3A), but only when the suckling stimulus *k_p_* is present; i.e., the modelled priming of dendritic release is essential. The model parameters were fine-tuned to match the interspike interval distributions of oxytocin cells (constructed both between bursts and within bursts) and the temporal characteristics of bursts (Fig. 3 and see [31]); these parameters were then fixed (Table 1). With these parameters, bursts comprise 50–70 spikes in 1–3 s (0.9–4.6 s *in vivo* [7]), and recur at intervals of ∼4 min (248 (48) s, mean (SD), range 149–388 s, based on 120 bursts), in close agreement with *in vivo* observations [5], [7], [8], [31]–[34].

The interspike interval histograms constructed between bursts match *in vivo* data indistinguishably [31] (Fig. 3B), confirming that the model accounts well for the background stochastic activity of the oxytocin cells, as well as bursting activity. Normally, all cells participate in the reflex in the model, with bursts approximately synchronized through the population. The mean variation in burst onset is 204±14 ms (mean±S.E. of 17 bursts), close to measurements *in vivo* (e.g. [5] reports delays of 0–386 ms between bursts in pairs of simultaneously recorded cells). Model neurons display a brief period of silence *preceding* many bursts; this feature mainly reflects the inhibitory actions of endocannabinoids; in the model, endocannabinoids released from the first cells that display a burst can suppress synaptic input enough to cause a brief inhibition in other oxytocin cells before they are activated by oxytocin release (Fig. 3D). Similar pre-burst silences occur *in vivo* (Fig. 3D red trace, and [10]).

In the model, the shape of the bursts is critically determined by the AHP mechanism, which reduces the peak firing rate and shortens the burst duration. Removing the AHP (by setting *k_AHP_* = 0) does not abolish bursting, and has little effect on the timing of bursts (data not shown), as it activated relatively little at the background firing rates. The HAP mechanism does affect the timing of bursts as it limits the occurrence of short interspike intervals; as an increase in the frequency of short intervals increases the rate of depletion of dendritic oxytocin but also increases the frequency of events that can trigger a burst, the effects of changing the HAP are complex. In the model the HAP was fixed to provide a good match to the interburst interspike interval distribution, and so the effects of varying this were not studied systematically. As well as an HAP, some oxytocin cells show a depolarising afterpotential, which may further facilitate bursting; in the present model we have neglected this as it is present in only a minority of oxytocin cells, and has no clear contribution to background firing patterns *in vivo* [32].

### Pacemaker versus Emergent Activity and Post Bursting Activity

**Figure 4:**
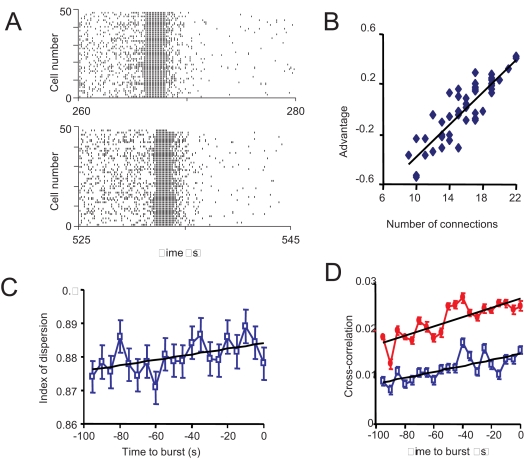
Random Onset of Bursts. (A) Raster plots showing the spikes generated by all the cells of the model in two bursts. (B) We considered a network with the same number of cells and bundles (and the same mean connectivity, ) but where the number of dendrites varied in each bundle. For each cell, the leader/follower character was measured by its mean ‘advantage’. The mean ‘advantage’ (start time relative to other cells, averaged over 120 bursts), plotted for each cell against the number of dendro-dendritic connections, shows that bursts are more likely to start in regions of the network where dendritic bundling is more pronounced. (C) The index of dispersion of the firing rate before bursts (in spikes/0.5 s, averaged over 5-s intervals; each point is an average over all cells and 136 bursts). The increase shows that firing is increasingly irregular just before a burst. The index of dispersion is the ratio of the SD of the firing rate to the mean firing rate over the same period. In the absence of retrograde attenuation by endocannabinoids (i.e. when α is set to 0), there is no increase in the index of dispersion, so the increased variability reflects the increasing antagonism between the excitatory effects of oxytocin and the inhibitory effects of endocannabinoids. (D) The cross-correlation of firing rates before bursts (in spikes/0.5 s; cross-correlations measured over 5 s-intervals, with zero time lag; average over 136 bursts; bars are S.E.M.). The blue squares are means of the cross-correlations between all pairs of cells in the network; the red circles are the mean ‘intra-bundle’ cross-correlations (the mean cross-correlation of all the pairs of cells projecting to the same bundle).

As observed *in vivo* [5] we found no fixed ‘leader’ or ‘follower’ cells, and the order in which neurons start to burst varies randomly with each burst (Fig. 4A). Thus bursting in the model is an emergent activity due to the interplay between the single neuron dynamics and network dynamics. The lack of a marked leader/follower character of the model neurons might have been accentuated by the homogeneous arrangement of the connections in the network used for simulations, as all bundles contained the same number of dendrites (*d_i_* = *d̅*, *i* = 1,…,*n_b_*). Therefore, we also considered a network with the same number of cells and bundles (and the same mean connectivity *d̅*) but where the number of dendrites varied in each bundle. For each cell, the leader/follower character was measured by its mean ‘advantage’
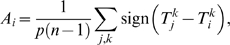
(12)where 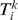 denotes the time of the onset of the *k*th burst in cell *i*, and *p* is the total number of bursts. *A_i_* is strongly correlated with the number of dendritic connections (r = 0.8796, *P* = 10^−16^; Fig. 4B). Thus bursts are more likely to start in regions of the network where dendritic bundling is more pronounced.

With no suckling input, the firing of oxytocin cells in the model is uncorrelated (as *in vivo*), as each receives a wholly independent synaptic input. Between bursts, spiking activity in the network is characterised by small but increasing cross-correlation of firing rates (Fig. 4C), a consequence of the strengthening of the interactions between cells. The background spike activity becomes progressively more irregular approaching a burst, as indicated by an increasing index of dispersion of the firing rate (Fig. 4D). Both results are in agreement with experimental findings *in vivo* [34]–[36]. In the model the increased variability arises because, towards a burst, activity produces dendritic oxytocin release, with excitatory consequences, but also endocannabinoid production, with inhibitory consequences but with different timescales; if endocannabinoid release is eliminated (by setting *α* = 0) then there is no increase in variability.

**Figure 5:**
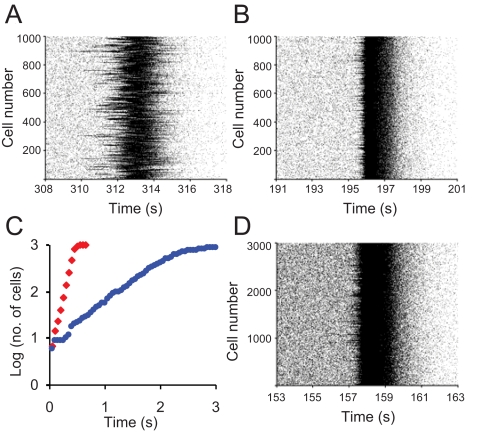
Network Connectivity and Co-ordination of Bursting Raster Plot of Spike Activity During a Burst in a Network of 1000 Neurons with (A) and (B). (C) The number of neurons recruited into bursting versus time after the first burst seen in the network, for a network of 1000 neurons with different connectivity (blue symbols, red symbols). The y axis is the logarithm of the number of recruited cells, so an exponential (“increasingly rapid”) growth appears as linear. Synchronisation occurs more rapidly when the connectivity is greater. (D) Raster plot of the spike activity during a burst in a network of 3000 neurons with.

We observed bursting in networks with varying number of neurons and/or bundles. In a network of 1000 neurons with limited bundling (*d̅* = 2), bursts occur rarely, propagate slowly, and involve only some cells (Fig. 5A). Increasing the degree of bundling, i.e. decreasing *n_b_*, leads to faster propagation and better synchronization (Fig. 5B). Figure 5C shows the propagation of a burst by plotting the temporal course of the number of cells recruited into a burst. The burst ‘wavefront’ grows exponentially with time, implying that even large networks can be rapidly synchronized. An example is given in Fig. 5D where we show a synchronized burst occurring in a network of 3000 neurons (*d̅* = 8).

**Figure 6:**
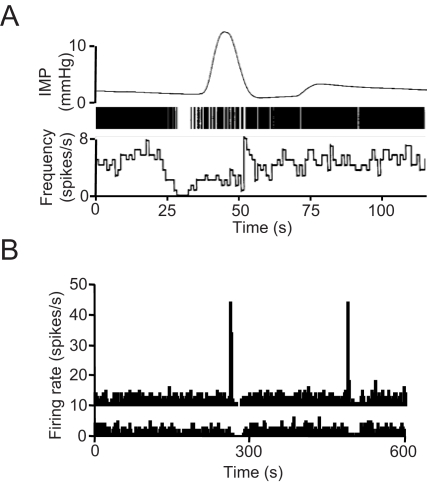
“Post-burst” Silences Observed in the Absence of Bursts. (A) The top trace shows the typical intramammary pressure response indicative of a reflex milk let-down in a lactating rat; the middle trace is a raster plot indicating the corresponding spike discharge of a supraoptic neuron, and the lower trace is the corresponding firing rate record. This cell showed no burst activity preceding milk ejection, but showed a typical “post-burst” silence. (Note that the increase in intramammary pressure normally occurs about 12 s after the milk-ejection burst; this delay reflects the delay in oxytocin released from the pituitary gland reaching the mammary gland, not a delay in oxytocin release). (B) Simultaneous activity of two cells in the model, in one of which the sensitivity to oxytocin has been disabled. While the upper cell shows typical intermittent bursts, the lower cell shows post-burst silences, but no bursts, due to removal of afferent excitation by oxytocin.

The bursts are followed by long silent periods (up to 20 s). *In vivo* [7] the post-burst inhibition is the most variable component of the burst, both in duration (7–56 s) and intensity, indicating that it is not simply the deterministic consequence of an activity-dependent AHP. In the model, the post burst silence is mainly a consequence of the prolonged suppression of afferent input, following the increase in endocannabinoid concentration after a burst. *In vivo*, some otherwise typical oxytocin cells have been observed occasionally which show no bursts at milk ejection but instead fall silent (Fig. 6A). A similar phenomenon can be replicated in the model by assuming that some neurons do not express oxytocin receptors (i.e. by setting *k_OT_*  = 0 for these neurons, Fig. 6B).

### Dendritic Storage and “Priming”

**Figure 7:**
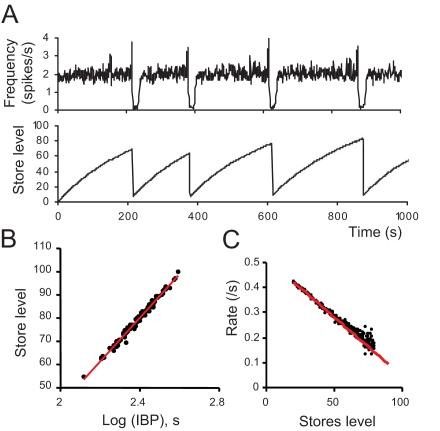
Role of Dendritic Release in Generating Bursts. (A) Upper trace: The evolution of the mean firing rate in the model (in spikes/s; average over all neurons); the vertical axis has been capped to highlight the fluctuations of the basal activity. Bottom trace: the evolution of dendritic stores level, given as the average over all the dendrites in the network; grey bars are SD. (B) The stores level at the time of the bursts (average over all dendrites) plotted against the logarithm of the inter-burst interval. (C) The rate of change of the stores plotted against the average store level. Both quantities are averaged over all dendrites. Mono-exponential behaviour, as expected from a process approaching saturation, would appear as a downward straight line, the slope being proportional to the average release rate from stores. This plot shows a slight departure from a mono-exponential trend at high stores levels, where there is a small decrease in the slope, corresponding to a reduction of the release rate, due to endocannabinoid release.

In the model, the dendritic stores of readily-releasable vesicles are continuously incremented by the suckling-related ‘priming’ input. Their level, averaged over the entire network, increases relatively steadily between bursts despite activity-dependent depletion (Fig. 7A), and bursts tend to occur when the stores are relatively large. The mean level at the time of bursts correlates strongly with the logarithm of the inter-burst interval (*r* = 0.99; *P*<10^−9^; Fig. 7B). Fig. 7C plots the rate of change of the stores against the store level (both averaged over the network). The decrease in slope at high levels reflects a reduction of the average release rate, and is a consequence of the suppression of afferent input as a result of endocannabinoid release. This stops the release from becoming regenerative, and allows the stores to increase further. In this phase, the network activity becomes more irregular because of the opposing feedback mechanisms: local activity-dependent excitation through the effects of dendritic oxytocin release, and inhibition due to suppression of afferent input. When the stores are large, spatially coordinated fluctuations of release can have a large impact on the dynamics. If just a few neighbouring cells show coincidentally increased activity due to stochastic variation in their input rates, and have large enough stores, then enough oxytocin can be released to trigger positive feedback and start a burst.

### Stability

**Figure 8:**
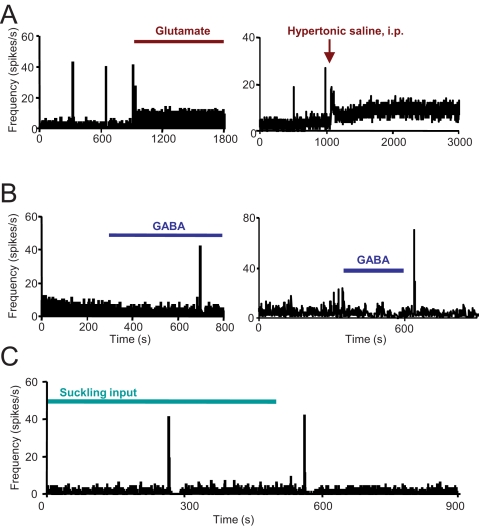
Paradoxical Behaviour, Observed Experimentally, Reproduced in the Model. (A) Left trace: A large increase in excitatory input rate will stop ongoing bursting activity in the model network. The bar marked ‘Glutamate’ corresponds to a 150% step increase in the excitatory input rate (from basal levels Hz; Hz). Right: Similarly, excitatory stimuli, such as systemic injection of hypertonic saline block ongoing bursting in oxytocin cells in vivo; from [36]. (B) Left: increasing the inhibitory synaptic input can paradoxically start bursting activity in the model when the suckling input is sub-threshold (kp = 1.4/s). The bar marked ‘GABA’ corresponds to a 150% step increase in the inhibitory input rate (from a basal level of 80 Hz). Right: The effect of local application of a GABA agonist to an oxytocin cell recorded from the supraoptic nucleus in vivo, from [36]. In the experiments, GABA was applied by local pressure injection; the timing of applications is marked, but the resulting exposure to GABA exceeds this, as evident here by the sustained reduction in background firing rate. Thus the burst occurs during elevated GABA exposure. (C) Firing rate of a model cell showing bursting in response to suckling input (bar). Note that, in this rare example, a single burst occurs after removing the suckling stimulus.

Increased spike activity between the bursts enhances depletion of the stores and so can delay or even suppress bursting (Fig. 8A); conversely, an increase in inhibitory input can promote the reflex in a system which fails to express bursting because of an insufficient priming (Fig. 8B). Such “paradoxical” behaviours have been extensively described *in vivo*; for example, injections of the inhibitory neurotransmitter GABA into the supraoptic nucleus of a suckled, lactating rat can trigger milk-ejection bursts [37] (Fig. 8B right); conversely, many stimuli that activate oxytocin cells, including the systemic administration of cholecystokinin, relaxin, or hypertonic saline, all suppress the reflex [e.g. 37] (Fig. 8A right). Very occasionally a single burst can occur shortly *after* removing the suckling stimulus (Fig. 8C). This feature is also shared (very occasionally) by the reflex *in vivo*, and indicates that suckling itself is not a strictly necessary trigger.

### Effects of Endocannabinoids

**Figure 9:**
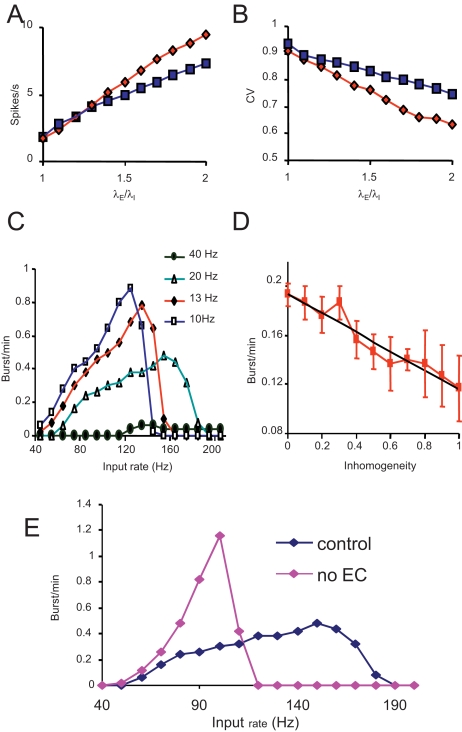
Effect of Suckling on Electrical Activity of Model Cells A and B. In the model, suckling results in activity-dependent retrograde inhibition of the synaptic inputs. Accordingly, as synaptic input level increases, electrical activity increases faster in the absence of suckling than in its presence. (A) The blue squares show the mean firing rate of model cells with normal network interaction (i.e. with suckling input), as a function of the mean synaptic input rate. The red diamonds show the behaviour in the same conditions but without suckling. The inhibitory input is fixed at 80 Hz. Thus in the model, suckling reduces the background activity of the fastest firing cells. (B) As in (A), but plotting the coefficient of variation (CV) of the interspike intervals (SD (interval)/mean (interval)). This standard measure of the irregularity of firing shows that the model cells fire more regularly as the mean level of synaptic input increases. The effect of suckling is to increase the irregularity of firing of neurons, mainly by reducing the firing rate of the fastest cells. (C) In the model, during suckling, the frequency of milk-ejection bursts is related to the average level of synaptic input in a biphasic manner. At very low and at very high levels of input, bursting will not occur. The frequency of bursts was obtained by simulating the model at varying excitatory input rates and, also shows the effect of altering the frequency threshold for dendritic release: the higher the threshold, the fewer bursts will occur. (D) The frequency of bursting depends on how homogeneous the background firing rate of oxytocin cells is. Here, we looked at the effect of a spatially inhomogeneous input on bursting frequency (mean of five trials of 50 min). Homogeneity is measured as the ratio of the SD of the synaptic input rate over the mean. The bars are SD. (E)The effect of endocannabinoids in the model is to increase the range of synaptic input rates compatible with bursting, and to make the mean rate at which bursts occur relatively independent of synaptic input rate within this range. Here, this is illustrated by looking at the consequences of removing the effect of endocannabinoids (no EC, by = 0). This is true for all the threshold values considered, the α setting panel shows the comparison for the control case (fth = 20 Hz).

Figure 9 shows the response of a model neuron in absence of suckling input; in this case, cells increase their mean firing rate more strongly in response to an increase of the excitatory input. In the model, during suckling, neurons that are strongly excited produce endocannabinoids that reduce the overall input level. This negative feedback defends the system from over-excitation, and maintains the network activity in an optimal range for bursting. This is an important feature, because bursting in the model is possible only within a range of values of excitatory input (Fig. 9C). The exact range depends on the strength of the coupling between spike activity and dendritic secretion (as measured by the frequency threshold for release *f_rel_* = 1/*τ_rel_*). At a low level of excitation, an increase in the excitatory rate favours bursting by increasing the frequency of release episodes which can trigger a burst. However, beyond a critical level, release events may be so frequent that stores are not replenished fast enough to reach the critical level required to trigger a burst. Under such conditions, bursts become rarer and less predictable, until eventually over-excitation disrupts the reflex.

As illustrated in Fig. 9E, the inhibitory effect of endocannabinoids reduces the likelihood of a burst being triggered at low synaptic rates, but also reduces the rate of depletion of dendritic oxytocin, thus increasing the probability of bursting at high synaptic input rates. The overall effect is to increase the range of synaptic input rates compatible with bursting, and to make the mean rate at which bursts occur relatively independent of synaptic input rate within this range.

Spatial inhomogeneity in the stochastic input can also degrade the reflex (Fig. 9D). With increasing spatial inhomogeneity, for a given average firing rate, there are more faster firing cells, and also more slowly firing cells. The faster firing cells will generate more short intervals – potential burst triggering events - but those events will be less potent because of greater depletion of their stores. For these events to trigger bursts, they must recruit responses from other cells to which they are connected – but the slower firing cells are less excitable (although they have higher store levels). The net result is that bursts are triggered less often. Thus the system performs optimally when the activity is relatively homogeneous between oxytocin cells, a conclusion previously drawn from experimental studies [33].

## Discussion

During lactation, oxytocin is released in pulses following quasi-synchronous bursts of electrical activity in oxytocin cells. Here, we showed that such bursting can arise as an emergent property of a spiking neuronal network. Our model does not incorporate all elements of the physiology of oxytocin cells, but finds a minimalist representation congruent with physiological evidence to help identify the key processes. We suggest that, during lactation, the oxytocin system is organized as a network where neurons interact by dendritic release of oxytocin coupled non-linearly to electrical activity. This requires a stimulus-dependent process of priming of the dendritic stores, whereby these are made available for activity-dependent release. Dendritic release of oxytocin occurs only when the neuron's firing rate is sufficiently large, so interactions between neurons are rare and erratic between bursts and in the absence of the suckling stimulus, leading to asynchronous spiking except during the bursts themselves; the network is essentially thus a pulse-coupled network.

The most distinctive features of our model are the increase of excitability as a consequence of priming, and the inhibition following the bursts; the inhibition is attributed here to endocannabinoids, but is also due in part to other retrograde messengers. Dendritic peptide release, which is likely to occur widely throughout the brain, is a key feature in the control of information transfer in neural networks, through cross-talk and autocontrol by paracrine/autocrine mechanisms.

Peptides are a large and diverse class of signalling molecules, and many different peptides are expressed in different neuronal populations. It has been argued elsewhere that some peptide signals are ‘broadcast’ throughout the brain by diffusional “volume transmission”, rather than by temporally and spatially precise synaptic transmission [38]. Hypothalamic neurons which release the same hormone are generally ‘tied together’ by means of autoreceptors for the peptides they produce; thus small amounts of peptides released locally ‘bind’ a population of neurons into co-ordinated activity, allowing the population to develop a synchronous burst that can initiate a wave of secretion that travels to more distant sites in the brain.

In the present model, bursting arises as an emergent behaviour of a very sparsely connected population of neurons. Bursting can begin at any of many foci of neuronal interactions – within any of the dendritic bundles that link just a few of the neurons, from where it will spread rapidly through the remaining bundles. Bursting arises by a positive feedback mechanism through activity-dependent release of oxytocin, the magnitude of which is down-regulated after a burst (by depletion of a pool of releasable oxytocin); the core mechanism is thus analogous to a mechanism used in some other models of bursting – positive feedback followed by synaptic depression. The topology of the networks is very different – the present network is very sparsely connected compared to others (e.g. [39],[40]), and the biological substrate is different – here the intercommunication is dendrodendritic rather than synaptic.

The model makes apparent sense of the role in the milk-ejection reflex of several biological phenomena. First, the afterhyperpolarisation, a slow activity dependent conductance, has a role only in shaping the burst profile; it contributes little to burst timing or to post-burst silences. Second, although the core mechanism inducing bursts is activity-dependent positive feedback, via release of oxytocin, negative feedbacks are also important. In the real system there are multiple negative feedback mechanisms involving several signalling molecules, here these are represented by only one – the production of endocannabinoids. In the model, endocannabinoid production is proportional to oxytocin release – a simplification, as the real determining factor is probably intracellular [Ca^2+^]. Importantly, the dynamics of the effects of endocannabinoids differ from those of oxytocin, and the dual effects promote increased variability in firing rate as the system swings from excitation to inhibition. The “upswings” mean that, for a given mean firing rate, there are more clusters of short intervals towards the end of an interburst interval, and they are more likely to be correlated between neurons, making them more potent as potential burst-triggering events. At the same time, the depressive effects on firing rate means that at high synaptic input rates there is less depletion of the releasable pool of oxytocin. Accordingly, the rate at which bursts arise is relatively independent of synaptic input rate over a reasonably wide range.

### Bursting, Spiking, and Multiscale Dynamics

Whereas neurons exchange information mostly via spikes, endocrine cells rely on hormonal pulses to signal to their target tissues. For many neurons, clustered spike activity can be optimally effective in inducing the required changes on the targets, but for endocrine cells to generate a signal large enough to be read at a distance, their secretory activity must not only be optimal for each cell, their activity must also be co-ordinated; hence peptide hormone signals are generally pulsatile [41]. Gonadotrophin-releasing hormone (GnRH) neurons also display synchronised bursts, possibly as a result of direct positive feedback from GnRH release [42]. Neuroendocrine cells are perhaps a special case in generating a classical hormone signal by co-ordinated electrical activity. However, many populations of neurons in the brain produce a peptide product as well as a conventional transmitter, and many of these peptides have effects on organismal behaviour that are hormone-like [17],[43], in that they act at dispersed and often distant targets to produce prolonged organisational changes. For a hormone-like, pulsatile signal to be produced reliably, the activity of a population of peptide-secreting neurons must be co-ordinated in a physiologically plastic manner. Such co-ordinated signals, coming from the individual nodes of an interactive network, must emerge from the dynamics at the lower level of organization (for the neuron case, from the dynamics of stochastic ionic channels coupled via the membrane potential). In the present model, network interactions are solely mediated by spikes with interspike intervals less than *τ_rel_*; similar spike *doublets* are thought to play a critical role in the synchronization of network activity in many neural systems [44]–[46].

### Limitations of the Model

The present model clearly produces a close match to electrophysiological data at the level of spike output, and its main strength is the simplicity of the representation of a single neuron; this makes it feasible to use the model to explore how properties of the network (connectivity and dynamics of intercommunication) affect the system behaviour. We believe that the simplifications are unlikely to have had any major influence, with two possible exceptions. First, we have not included intracellular [Ca^2+^] as a variable, although mobilisation of intracellular Ca^2+^ can trigger dendritic oxytocin release, and therefore probably contributes to the central oxytocin release during milk-ejection. Implicitly we have assumed that this overlaps with activity-induced oxytocin release and can be neglected, but it is possible that in some circumstances oxytocin release triggered by Ca^2+^ release from intracellular stores might precipitate a burst. Second, we modelled dendritic release as a relatively common deterministic event – small packets released fairly frequently. Dendritic release probably involves the relatively rare exocytosis of large vesicles that each contains a very large amount of oxytocin – and release is likely to be highly stochastic, with interval length governing the probability of release rather than determining it. Whether this will affect the model behaviour substantially remains to be tested.

## Supporting Information

Video S1 Spikes (pink) and oxytocin release (red) in a neuronal network model of the milk-ejection reflex.
(4.23 MB AVI)
